# Dataset about the Life Cycle Assessment of new fermented food products mixing cow milk and pea protein sources

**DOI:** 10.1016/j.dib.2023.109263

**Published:** 2023-05-24

**Authors:** Juliette Huguet, Christophe Chassard, René Lavigne, Françoise Irlinger, Isabelle Souchon, Stephan Marette, Anne Saint-Eve, Caroline Pénicaud

**Affiliations:** a Université Paris-Saclay, INRAE, AgroParisTech, UMR SayFood, Palaiseau 91120, France; b Université Clermont Auvergne, INRAE, Vetagro Sup, UMRF, Aurillac 15000, France; c Avignon Université, INRAE, UMR SQPOV, Avignon 84000, France; d Université Paris-Saclay, INRAE, AgroParisTech, Paris-Saclay Applied Economics, Palaiseau 91120, France

**Keywords:** Inventory data, Environmental impacts, Environmental performance, Sustainability, Sustainable proteins, Legumes, Dairy products

## Abstract

In recent years, the food industry has expended considerable effort to design novel products that replace animal proteins with legumes; however, the actual environmental benefits of such products are often not quantified. Here, we performed Life Cycle Assessments (LCA) to evaluate the environmental performance of four new fermented food products based on different mixtures of animal (cow milk) and plant (pea) protein sources (100% Pea, 75% Pea-25% Milk, 50% Pea-50% Milk, 25% Pea-75% Milk). The system perimeter encompassed all stages from agricultural production of the ingredients to the creation of the final ready-to-eat products. Impacts were calculated for all environmental indicators included in the EF 3.0 Method in SimaPro software based on a functional unit of 1 kg of ready-to-eat product. Life cycle inventories included all of the flows analyzed by the LCA (raw materials, energy, water, cleaning products, packaging, transport, waste). Foreground data were acquired directly on the manufacturing site; background data were taken from the Ecoinvent 3.6 database. The dataset contains details on the products, processes, equipment, and infrastructure considered; mass and energy flows; Life Cycle Inventories (LCI); and Life Cycle Impact Assessment (LCIA). These data improve our understanding of the environmental impact of plant-based alternatives to dairy products, which is currently poorly documented.

**Specification Table**

**Table:** 

Subject		Environmental Science
Specific subject area		Environmental assessment of four new fermented food products based on different mixtures of animal (cow milk) and plant (pea) protein sources (100% Pea, 75% Pea-25% Milk, 50% Pea-50% Milk, 25% Pea-75% Milk, w/w)
Type of data		Table
How data were acquired		Inventory data were obtained by manual measurement or calculation, or were found in the technical and scientific literature. Background data mainly come from the database Ecoinvent 3.6. Life Cycle Assessments were computed using SimaPro 9.1.0.11 software and the method “EF 3.0 Method (adapted) V1.00 / EF 3.0 normalization and weighting set”.
Data format		Raw Analyzed
Description of data collection		The dataset contains LCIs and LCIAs of four new fermented food products based on different mixtures (ratios) of animal (cow milk) and plant (pea) protein sources. LCI data were collected during experiments performed in September 2019 at an experimental technology platform facility in France, as well as from equipment data sheets. Calculations were performed to quantify energy flows. Scientific literature and databases were also used as sources of data. Results were calculated for 1 kg of each final product using the EF 3.0 method.
Data source location	Institution	INRAE
	City/Town/Region	Aurillac (LCI data) and Palaiseau (LCIA data)
	Country	France
Data accessibility	Repository name	Data INRAE
	Data identification number	https://doi.org/10.57745/X4QWKZ
	Direct URL to data	https://doi.org/10.57745/X4QWKZ
	Related research article	Huguet, J., Chassard, C., Lavigne, R., Irlinger, F., Souchon, I., Marette, S., Saint-Eve, A., Pénicaud, C. Environmental performance of mixed animal and plant protein sources for designing new fermented foods, Cleaner Environmental Systems, 9 (2023) 100115, 10.1016/j.cesys.2023.100115.

## Value of the Data

• This dataset presents LCIs (Life Cycle Inventories) and LCIAs (Life Cycle Impact Assessments) of four new fermented food products based on different mixtures of animal (milk) and plant (pea) protein sources.
• The Life Cycle Inventory data and Life Cycle Impact data in this dataset ensure transparency in the LCA modeling described in the accompanying article [1].
• The presented experimental setups and LCA methodology can serve as an orientation for further scientific work on this topic.
• The Life Cycle Inventory data could be reused by scientists for future LCAs.
• These data can be used to support assessments of sustainability in the food sector.
• These data can be used to inform design recommendations for the production of more environmentally friendly foods.

## 1 Objective

There is a lack of data on the environmental impacts of plant-based alternatives to cheese. We generated this dataset to fill this gap, by quantifying the environmental impacts associated to the production of new fermented products alternatives to cheese, created using different combinations of plant (pea) and animal (cow milk) protein sources. The new fermented products were all created using a cheese-making process (Camembert production) with pilot equipment that mimicked semi-artisanal production at the facility of a technology platform in Aurillac, France. These data were used in a research article in order to: (i) identify the environmental hotspots of the production process; (ii) compare the environmental performance of different mixes; (iii) compare the environmental performance of the studied products with that of Camembert (same manufacturing technology as the new products), hummus and tofu (two other plant-based products).

The present data paper provides the data collected during the production at the facility of the technology platform and the LCA results. This brings transparency to the associated research article. The data are sufficiently described with metadata to be reused by other scientists willing to complete their own data with such information.

## 2 Data Description

The dataset associated with this article (10.57745/X4QWKZ) contains the inventory data related to four fermented food products based on different mixtures of animal and plant protein sources (data on ingredients, processing, and transport) and the LCIA results presented in the associated article [1]:

1. LCI_fermented_products data: Life Cycle Inventory for the four fermented products. All the mass and energy flows are indicated, grouped by step and sub-step of the production process. The measured flows refer to the total mass of the final product. These data need to be combined with information from the LCI_parameters dataset to obtain the exact LCI flows for each food product. The names of all the data used for the inventories and the associated database are also given. Some comments are added to help understand the meaning of the formulas describing the amounts of data.
2. LCI_parameters data: All complementary parameters needed to complete the LCI_fermented_products data (Pea_ratio, Milk_Ratio, Nb_molds, Losses_molding, Losses_demolding, Mass_product, Total_mass_final_product).
3. LCI_others: Life Cycle Inventories of frozen lactic acid bacteria, obtained from Ref. [2] and adjusted based on the data used and the associated database. This file also contains the Life Cycle Inventory of household waste used in this study, together with the name of the data used and the associated database.
4. LCIA_fermented_products: Life Cycle Impact Assessment results obtained for 1 kg of each ready-to-eat product for all the environmental indicators evaluated by the “EF 3.0 Method (adapted) V1.00 / EF 3.0 normalization and weighting set”: Climate change, Ozone depletion, Ionizing radiation, Photochemical ozone formation, Particulate matter, Human toxicity (non-cancer), Human toxicity (cancer), Acidification, Eutrophication (freshwater), Eutrophication (marine), Eutrophication (terrestrial), Ecotoxicity (freshwater), Land use, Water use, Resource use (fossils), Resource use (mineral and metals). Results are given by sub-step of the process as well as in total for each indicator.

**Fig. 1:**
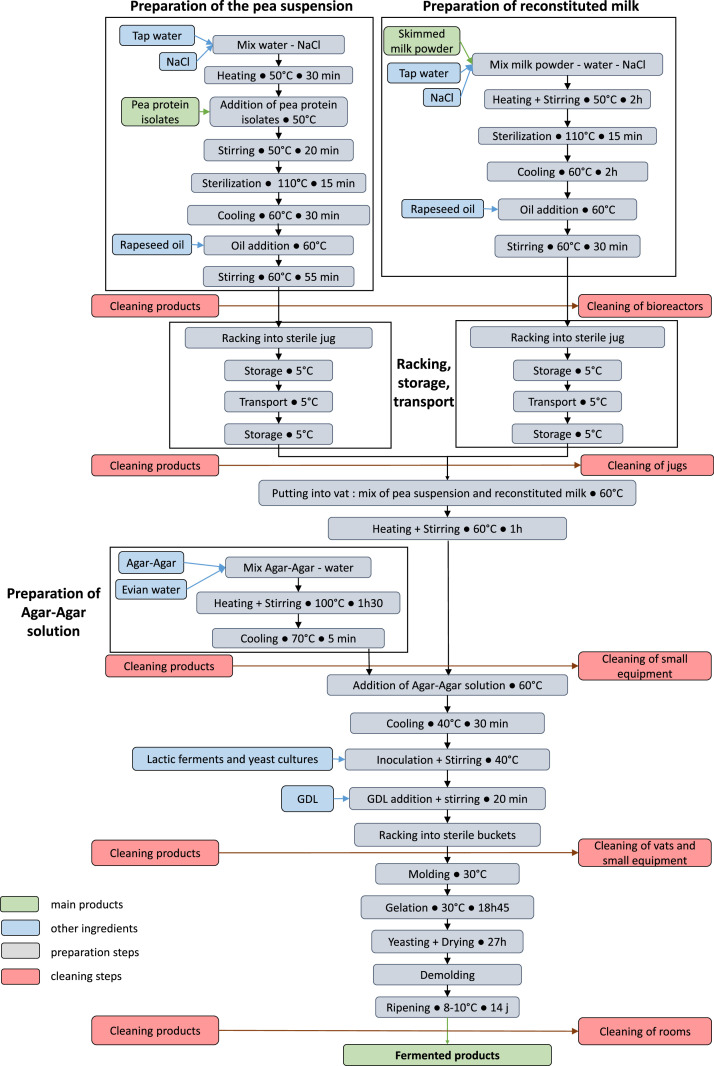
Manufacturing process of the new fermented food products based on mixtures of animal and plant protein sources.

Complementary, in the body of the present data paper, Fig. 1 presents the detailed manufacturing process of the new fermented food products based on mixtures of animal and plant protein sources. Tables 1 and 2 show the compositions of the pea and reconstituted milk suspensions, respectively. Table 3 displays the composition of the mixtures produced by combining pea suspension and reconstituted milk. Table 4 provides information on equipment used for the manufacturing of new fermented food products that mix animal and plant protein sources.

**Table 1:** Composition of the pea suspension.

Ingredients	Amounts (kg)
Water	64
Pea protein isolates	11.4
Rapeseed oil	7
Salt	0.19

**Table 2:** Composition of the reconstituted milk suspension.

Ingredients	Amounts (kg)
Water	32.4
Skimmed milk powder	15.43
Rapeseed oil	4.7
Salt	0.132

**Table 3:** Composition of the mixtures produced by combining pea suspension and reconstituted milk.

	Pea suspension (kg)	Reconstituted milk (kg)
100% Pea	29	0
75% Pea	21.75	7.25
50% Pea	14.5	14.5
25% Pea	7.25	21.75

**Table 4:** Equipment used for the manufacturing of new fermented food products that mix animal and plant protein sources.

	Material	
Equipment	Nature	Unit mass (kg)
Bioreactor pea	Stainless steel	480
Bioreactor milk	Stainless steel	350
Autoclave 1	Stainless steel	500
Autoclave 2	Stainless steel	550
Preparation vat	Stainless steel	49
Grid shelves	Stainless steel	21
Bucket	Stainless steel	1.16
Bottle	Glass	1.43
Jug	Polypropylene	1.94
Pitcher	Polyethylene	0.297
Mat	Polyethylene	0.12
Mold	Aluminum	0.00288

## 3 Experimental Design, Materials and Methods

### 3.1 Production System of Fermented Food Products

The production system of the fermented food products is depicted in Fig. 1 and detailed below.

### 3.2 Ingredient Production

Data regarding the production of raw ingredients were obtained from the LCI databases Ecoinvent 3.6 and Agribalyse 3.0 (for each ingredient, the database of origin is indicated in the LCI_fermented_products file). This included the agricultural stages necessary to produce the ingredients.

### 3.3 Preparation of the Pea Suspension

The first step of the manufacturing process was preparation of the pea suspension. This was carried out by the company Greencell, by mixing 64 kg of tap water with 190 g of NaCl (supplier VWR) in an 80 L stainless steel bioreactor (Biolafitte). The solution was heated to 50 °C for 30 min by means of a hot water circulation system in the bioreactor's double jacket. To this, 11.4 kg of pea protein isolate (NUTRALYS^Ⓡ^), obtained from Roquette (Lestrem, France), were added. The medium was stirred and maintained at 50 °C for 20 min at 1000 rpm. It was then sterilized *in situ via* steam injection at 110 °C for 15 min to eliminate all microorganisms present. The medium was maintained at 60 °C for 30 min, then 7 kg of rapeseed oil (Fleur de Colza, Lesieur, France) were added. Finally, the suspension was stirred for 55 min at 600 rpm at 60 °C. The composition of the pea suspension is summarized in Table 1.

### 3.4 Preparation of Reconstituted Milk

The reconstituted milk was also prepared by Greencell. For this, 15.43 kg of skimmed milk powder from Lactalis (Bourgbarré, France) were mixed with 32.4 kg of tap water and 132 g of NaCl (supplier VWR) in a 60 L stainless steel bioreactor (Pierre Guérin). The solution was heated to 50 °C by the circulation of hot water in the double jacket, and homogenized for 2 h at 621 rpm. The reconstituted milk was then sterilized in the bioreactor by steam injection at 110 °C for 15 min. The medium was cooled for 2 h to reach a temperature of 60 °C. Then, 4.7 kg of rapeseed oil (Fleur de Colza, Lesieur, France) were added to the reconstituted milk. The mixture was stirred for 30 min at 621 rpm and maintained at 60 °C. The composition of the reconstituted milk suspension is summarized in Table 2.

### 3.5 Racking, Storage, and Transport of Preparations

The pea suspension and reconstituted milk were racked into sterile polypropylene jugs over the course of 10 min. The jugs were transported by refrigerated truck from Greencell to the Aurillac technology platform (85 km away), where they were stored in a cold room at 4 °C.

### 3.6 Putting into Vats, Heating, and Stirring

Once the preparations were made, the pea suspension and reconstituted milk were mixed directly in four 40 L stainless steel vats (Duchemin Frères) according to the desired proportions of pea suspension and milk (Table 3). The mixtures, of 29 kg each, were then heated to 60 °C with agitation (50 rpm) for 1 h.

### 3.7 Preparation of the Agar-Agar Solution

Agar-agar, a natural gelling polymer extracted from seaweed, was used as a texturizing agent. Agar-agar powder (290 g per tank) was mixed with Evian water (1.45 kg per tank) in 4 L Schott bottles. The solution was boiled (100 °C), with stirring, for 1 h30. The solution was then stirred at 50 rpm and cooled for 5 min until it reached 70 °C, forming a gel.

### 3.8 Addition of Agar-Agar Solution and Cooling

The prepared agar-agar solution was added to each preparation vat. The temperature was then lowered and maintained at 40 °C for 30 min before plating.

### 3.9 Inoculation

To the lukewarm (40 °C) matrices, 193 g of lactic ferments and yeast cultures were added per tank, with stirring (50 rpm). These were composed of lactic acid bacteria (*Lacticaseibacillus rhamnosus* and *Lactococcus lactis*) and yeast (*Kluyveromyces lactis* and *Geotrichum candidum*). Lactic acid bacteria transform the sugars present in the medium into organic acids, in particular lactic acid. This leads to acidification of the medium, which inhibits the proliferation of acid-sensitive pathogenic microorganisms. Instead, the yeasts consume the sugars and release ethanol and carbon dioxide. They also consume lactic acid to deacidify the environment. Together, these cultures enrich the nutritional and organoleptic qualities of the products.

### 3.10 Addition of GDL

GDL powder (glucono-delta-lactone) was added to each of the manufacturing vessels (145 g per vat), which were then maintained at 40 °C for 20 min, with stirring at 50 rpm. After dissolving in the medium, this molecule slowly hydrolyzes to form gluconic acid, which is converted to protons and gluconate. The release of protons decreases the electrostatic repulsion between the protein aggregates and increases the firmness of the gel. GDL thus acts as a gelling agent, improving the texture of products.

### 3.11 Filling and Molding

The four mixes were manually poured into 2 L stainless steel buckets, which were used to fill aluminum molds placed on carts. Approximately 165 individual products were molded per mix type.

### 3.12 Gelation

The molded products were placed in a temperature-controlled room at 30 °C and 95% relative humidity for 18 h and 45 min in order to facilitate their solidification.

### 3.13 Yeasting and Drying

The products were then air-dried for 27 h in the temperature-controlled room at an initial temperature of 25 °C and a relative humidity of 95%. Every 3 h, the temperature was lowered by 5 °C until it reached 10 °C. These conditions were optimal for the development of yeast on the surface of the products. The temperature and humidity were carefully controlled to promote core drying while avoiding the formation of cracks.

### 3.14 Unmolding

At the end of the yeasting and drying stage, the gels were carefully removed from the molds by hand and placed on flexible, fine-mesh polypropylene draining mats, which allowed the products to breathe and not stick to the surface. They were then ready for maturing.

### 3.15 Ripening

The products were placed in a ripening cellar at 9 °C and 92.5% relative humidity, which was carefully controlled to avoid the formation of crusts or cracks on the products’ surfaces. They were ripened in these conditions for 14 days. Ripening conditions have a crucial impact on the development of the ripening microbial community. During this period, the products undergo physico-chemical transformations through the action of natural and microbial enzymes, which gives them their organoleptic characteristics.

### 3.16 Cleaning

Before use, all materials were sterilized, either by *in-situ* sterilization (bioreactors, preparation vat) or using an autoclave (small equipment).

After use, the bioreactors were cleaned first using sodium hydroxide (30.5% w/w), then nitric acid (58% w/w) and Oxirisil. The bioreactor used to prepare the pea suspension was cleaned for 25 h30 and the bioreactor used to prepare reconstituted milk was cleaned for 23 h10.

After use, the preparation vat was cleaned for 60 min using sodium hydroxide solution (0.1% w/w).

Small items of equipment were cleaned after use with a dishwashing machine with the appropriate detergent (Sun^Ⓡ^). Three cycles of the dishwashing machine (90 min each cycle) were necessary to clean all the materials.

The rooms used for preparation, resting, and ripening were cleaned for 90 min with acidic foam (0.15% w/w).

## 4 LCA Methodology

Our study followed ISO standard 14040 [3].

### 4.1 Goal and Scope

Attributional LCA was performed, with two objectives: (i) to determine the impacts generated by the production process of four new fermented products, by identifying the steps that contribute the most to environmental degradation, and (ii) to use these data to compare the environmental performance among the four products and between these products and ones that are similar with respect to composition (hummus, tofu) or manufacturing technology (production of Camembert-type cheese).

The functional unit was 1 kg of ready-to-eat product.

The study took a “cradle to gate” approach: the scope of the system included all processes involved in the manufacture of each product, from the agricultural production of the ingredients to the creation of the final ready-to-eat product. However, packaging of the final products was excluded from the study due to a lack of appropriate data.

### 4.2 Life Cycle Inventory

The Life Cycle Inventories (LCI) of the products are presented in the table LCI_Fermented_Products. The flows measured for the LCI were for the total masses of product obtained at the end of the production process, i.e. after ripening. These masses are detailed for each product in the table LCI_parameters.

#### 4.2.1 Equipment

The equipment used is summarized in Table 4 together with the mass and nature of material that make up each item. These data were obtained from equipment data sheets or direct measurement when possible. It must be noted that, as a precaution, more jugs and mats were sterilized than were used for production. Also, the sterilization of all equipment for the four matrices was performed simultaneously in Autoclave 1.

These materials were modeled in the LCI using temporal allocations (Eq. (1)). The lifetime of all equipment was considered to be 30 years, except for the molds which were used only for this experiment (in this case, their lifetime was considered to be 45.6 h).

(1) $$Temporal\;allocation\;factor=\frac{Duration\;of\;use\;of\;the\;equipment\;\left(h\right)}{Lifetime\;of\;the\;equipment\;\left(h\right)}$$

#### 4.2.2 Infrastructure

We modeled the use of three different areas at the facility of the Aurillac technology platform: the preparation room, the resting room, and the ripening room. For each, we considered the resources consumed by the temperature control system: ethylene glycol, tap water, and electricity. These data were obtained from data sheets.

For these rooms, a temporal allocation was applied as described by Eq. (1). In addition, a volume allocation was used as follows:

- Preparation room: 1/4 of the room per matrix (the entire room was used; the four matrices were prepared at the same time);
- Resting room: 1/12 of the room per matrix (1/3 of the room was used in total; the four matrices rested at the same time);
- Ripening room: 1/8 of the room per matrix (1/2 of the room was used in total; the four matrices were ripened at the same time).

#### 4.2.3 Mass Flows

Mass flows of ingredients, water, cleaning products, product losses, and waste were either measured manually or estimated during the experiment.

The LCI of frozen lactic acid bacteria was obtained from a previous study [2].

Loss of refrigerant of Cold room 1 was calculated as in Ref. [2] (Eq. (2)).

(2) Lossofrefrigerant=Equipmentpower*Refrigerantcharge*Leakagerate

The equipment power and refrigerant charge were obtained from data sheets. The annual leakage rate of the refrigerant contained in the cold room was assumed to be 15% [4,5].

Packaging and mold waste were disposed of either by incineration (53%) or in a landfill (47%).

#### 4.2.4 Energy Flows

The electrical consumption (kWh) of a piece of equipment was calculated from its power *P* (kW) and the duration of use *t* (h) (Eq. (3)).

(3) Electricalconsumption=P*t

The energy necessary to produce steam *Q* (kJ) was obtained from theoretical calculations (Eq. (4)).

(4) Q=m*(ΔHv+(Cp*ΔT))

where, *m* is the mass of liquid water (kg), *ΔHv* the vaporization heat (2258 kJ/kg), *Cp* the specific heat of water (4.2 kJ/(kg.K)), and *ΔT* the difference in temperature to bring water to 100 °C (in this case 80 K).

#### 4.2.5 Transport

Only the transport of the pea suspension and reconstituted milk was included in the perimeter of the study. This transport (85 km) was performed by a refrigerated truck.

#### 4.2.6 Databases

The main database used was Ecoinvent 3.6; this was complemented by data from SimaPro on the substances involved in steam waste and by data from Agribalyse 3.0 on milk powder.

### 4.3 Impact Assessment

The Life Cycle Impact Assessments (LCIAs) were performed using SimaPro 9.1.0.11 software and the “EF 3.0 Method (adapted) V1.00 / EF 3.0 normalization and weighting set” [6]. All the midpoint impact categories available in this method were calculated: Climate change, Ozone depletion, Ionizing radiation, Photochemical ozone formation, Particulate matter, Human toxicity (non-cancer), Human toxicity (cancer), Acidification, Eutrophication (freshwater), Eutrophication, (marine), Eutrophication (terrestrial), Ecotoxicity (freshwater), Land use, Water use, Resource use (fossils), and Resource use (mineral and metals). Raw LCIA results were divided by the masses of the final products to obtain the values for the functional unit of 1 kg of final product. The LCIAs for the functional unit of 1 kg of final product are presented in the table LCIA_Fermented_Products.

## Ethics Statement

This work did not involve human subjects or laboratory animal, therefore did not meet any ethical issues.

## CRediT authorship contribution statement

**Juliette Huguet:** Investigation, Formal analysis, Methodology, Writing – original draft. **Christophe Chassard:** Conceptualization, Investigation, Methodology, Writing – review & editing. **René Lavigne:** Investigation, Methodology, Writing – review & editing. **Françoise Irlinger:** Conceptualization, Investigation, Methodology, Writing – review & editing. **Isabelle Souchon:** Conceptualization, Investigation, Methodology, Writing – review & editing. **Stephan Marette:** Conceptualization, Funding acquisition, Methodology, Writing – review & editing. **Anne Saint-Eve:** Conceptualization, Methodology, Writing – review & editing. **Caroline Pénicaud:** Conceptualization, Formal analysis, Funding acquisition, Investigation, Methodology, Supervision, Writing – review & editing.

## Declaration of Competing Interest

The authors declare that they have no known competing financial interests or personal relationships that could have appeared to influence the work reported in this paper.

## Data Availability

Life Cycle Assessment of new fermented food products mixing cow milk and pea protein sources (Original data) (Dataverse).
